# Solving the Independent Domination Problem by the Quantum Approximate Optimization Algorithm

**DOI:** 10.3390/e26121057

**Published:** 2024-12-05

**Authors:** Haoqian Pan, Changhong Lu

**Affiliations:** School of Mathematical Sciences, Key Laboratory of MEA (Ministry of Education) & Shanghai Key Laboratory of PMMP, East China Normal University, Shanghai 200241, China; chlu@math.ecnu.edu.cn

**Keywords:** quantum approximate optimization algorithm, independent domination problem, Qiskit

## Abstract

In the wake of quantum computing advancements and quantum algorithmic progress, quantum algorithms are increasingly being employed to address a myriad of combinatorial optimization problems. Among these, the Independent Domination Problem (IDP), a derivative of the Domination Problem, has practical implications in various real-world scenarios. Despite this, existing classical algorithms for the IDP are plagued by high computational complexity, and quantum algorithms have yet to tackle this challenge. This paper introduces a Quantum Approximate Optimization Algorithm (QAOA)-based approach to address the IDP. Utilizing IBM’s qasm_simulator, we have demonstrated the efficacy of the QAOA in solving the IDP under specific parameter settings, with a computational complexity that surpasses that of classical methods. Our findings offer a novel avenue for the resolution of the IDP.

## 1. Introduction

The Independent Domination Problem (IDP) seeks to identify the smallest independent dominating set (IDS) within a graph G(V,E). An IDS is defined as a subset D⊆V such that every vertex in V∖D is adjacent to at least one vertex in *D*, and no two vertices in *D* are adjacent. This problem is particularly relevant in practical applications, such as wireless network design. It has been firmly established that the IDP is NP-complete across numerous graph classes, including but not limited to chordal, bipartite, circle, and triangle graphs [1,2,3,4]. Due to its inherent computational complexity, research has increasingly focused on approximation and heuristic solutions tailored to specific graph structures, aiming to improve performance and feasibility. Notable advances include a polynomial-time algorithm with a complexity of $O(|V{|}^{2})$ for permutation graphs [5]. Similarly, for cocomparability graphs, a sophisticated algorithm has been proposed with a computational complexity of $O(|V{|}^{2.376})$, representing a significant optimization for this graph class [6]. Additional efforts have targeted at-most-cubic graphs, showcasing the adaptability of algorithmic approaches to diverse graph types [7]. For general graphs without specific structural constraints, recent work has produced exponential-time algorithms, including those with complexities of $2^{0.529|V|}$, $2^{0.441|V|}$, and $2^{0.424|V|}$, reflecting progress toward more efficient exponential solutions [8,9,10]. Furthermore, heuristic approaches such as memetic algorithms [11,12] and evolutionary algorithms [13] have gained traction in recent years. Despite these advancements, reducing the algorithmic complexity of the IDP remains a formidable challenge, driving ongoing research and development in this domain.

In recent years, advancements in quantum computing technologies [14,15,16] have significantly accelerated the application of quantum algorithms to address combinatorial optimization problems. Among these, the Quantum Approximate Optimization Algorithm (QAOA) [17] has emerged as a leading approach, demonstrating versatility across various combinatorial optimization challenges within the quantum computing domain. A range of problems have been explored using the QAOA on quantum hardware and simulators, including the Max-Cut problem [17], the Traveling Salesman Problem [18], the Domination Problem (DP) [19], the Minimum Vertex Cover Problem [20], the Boolean Satisfiability Problem [21], and the Graph Coloring Problem [22], among others. Beyond these application-specific studies, significant efforts have been devoted to improving the QAOA framework itself. For instance, Chandarana et al. [23] and Wurtz and Love [24] enhanced the convergence rate of the QAOA’s approximation ratio by incorporating a problem-dependent counterdiabatic driving term into the QAOA ansatz, effectively reducing the circuit depth. Similarly, Herrman et al. [25] extended the QAOA’s rotation angles by introducing vectorized parameters, leading to both increased approximation ratios and reduced quantum circuit depths, particularly for the Max-Cut problem. Chalupnik et al. [26] proposed adding a multi-parameter, problem-independent layer to the QAOA structure, achieving higher approximation ratios when solving Max-Cut on random regular graphs. Furthermore, Wang et al. [27] demonstrated improved performance by selectively optimizing quantum circuits while preserving the integrity of the objective function. Magann et al. [28,29] introduced two algorithms: FALQON and FALQON+. The former leverages qubit measurements in feedback-based quantum optimization to eliminate the need for classical optimizers, while the latter combines FALQON’s initialization with the QAOA to enhance parameter initialization. Additionally, Zhou et al. [30] addressed hardware constraints, such as the limited number of qubits on current quantum devices, by proposing the QAOA-in-QAOA method. This approach applies a divide-and-conquer strategy to partition a graph into smaller subgraphs, solving the Max-Cut problem on these subgraphs in parallel using the QAOA and subsequently combining the results with an overarching QAOA layer. This innovation enables the QAOA to tackle larger-scale problems even within the constraints of limited quantum resources. Further recent developments in the QAOA can be found in Blekos et al. [31]. Despite the extensive progress in quantum computing and QAOA research, a notable gap exists in the application of quantum algorithms to the IDP. While the QAOA has proven effective for various combinatorial optimization problems, its potential for addressing the IDP remains unexplored. Thus, the feasibility and effectiveness of the QAOA or other quantum approaches in solving the IDP remain open research questions, warranting further investigation.

The primary contribution of this paper lies in our pioneering application of the QAOA to solve the IDP, marking the first instance of utilizing a quantum algorithm for this specific challenge. We conducted comprehensive evaluations of the effectiveness of the QAOA in addressing the IDP, focusing on two key aspects: fundamental testing and robustness testing. The findings presented herein offer valuable insights and foundational experience that will inform and guide future endeavors aimed at solving the IDP using quantum computing technologies.

The structure of this paper is organized as follows: In Section 2, we systematically introduce the methodology for transforming the IDP into a 0–1 integer programming model, followed by the formulation of a Quadratic Unconstrained Binary Optimization (QUBO) model and its corresponding Hamiltonian. Section 3 presents an overview of the QAOA and delineates the procedural steps required to apply this algorithm for solving the IDP. Subsequently, in Section 4, we conduct both fundamental and robustness testing to assess the effectiveness of the QAOA-based algorithm in addressing the IDP. This section also includes a complexity analysis of the QAOA. Finally, Section 5 provides a comprehensive summary of the paper.

## 2. Problem Modeling

When using quantum algorithms for combinatorial optimization problems, a common approach is to model or transform the problem into a QUBO format, which is then mapped to the Hamiltonian of the Ising model. In this chapter, we formally define the IDP and derive its corresponding Hamiltonian representation.

### 2.1. Problem → 0–1 Integer Programming Model

Before exploring the IDP, we first define the dominating set (DS). Given a graph G=(V,E), a DS, *D*, is a subset of *V* such that for every vertex v∈V, the closed neighborhood N[v] intersects with *D* (i.e., N[v]∩D≠∅). Here, N[v] denotes the closed neighborhood of vertex *v*, comprising *v* and all vertices adjacent to it. The goal of the DP is to find the smallest such *D*. The IDP extends the DP by adding a constraint: *D* must also be independent, meaning no edges exist between vertices in G[D]. To enable the application of the QAOA for solving this problem, we first construct a mathematical model of the IDP in the following sections.

(1) $$\begin{array}{cc} \min_{\left\{X_{i}\right\}}\; & \sum_{i=1}^{\left|V\right|}X_{i} \end{array}$$

(2) $$\begin{array}{cc} s.t.\; & \sum_{j\in N[i]}X_{j}\geq 1\;\forall i\in V \end{array}$$

(3) $$\begin{array}{cc} & X_{i}\in \left\{0,1\right\}\;\forall i\in V \end{array}$$

(4) $$\begin{array}{cc} & X_{i}\cdot X_{j}=0\;\forall ij\in E \end{array}$$

In this model, $X_{i}$ is a binary variable, where $X_{i}=1$ indicates that vertex *i* is included in *D*, and $X_{i}=0$ otherwise. The expression $\sum_{i=1}^{\left|V\right|}X_{i}$ thus represents the total number of vertices in the DS. Our objective is to minimize |D|, mathematically expressed as $\sum_{i=1}^{\left|V\right|}X_{i}$. Constraint (2) ensures that for each vertex v∈V, either *v* itself or one of its neighbors is included in *D*, which is a fundamental requirement of the DP. Constraint (4), specific to the IDP, addresses the edges in *G*, categorized as follows: (1) edges within G[D], (2) edges within G[V∖D], and (3) edges connecting *D* to V∖D. For the second and third categories, Constraint (4) is automatically satisfied since at least one vertex in each edge has $X_{i}=0$. However, for edges within G[D], Constraint (4) requires that no edges exist, ensuring that the independence condition is met. At this stage, we have established the 0–1 integer programming model for the IDP. The next step is to transform this model into a QUBO formulation and subsequently convert the QUBO model into its Hamiltonian representation.

### 2.2. 0–1 Integer Programming Model → QUBO Model

The QUBO model is formulated in Equation (5), where *x* represents the vector of binary variables, and *Q* is the matrix of constants, commonly known as the QUBO matrix.

(5) $$minimize/maximize\;y=x^{t}Qx$$

Since the original objective function already satisfies the requirements of the QUBO model, our primary task at this stage is to convert the constraints into quadratic penalty terms and integrate them into the objective function. For each vertex i∈V, Constraint (2) can be expressed in the following general form:

(6) $$X_{1}+X_{2}+\cdots +X_{n}\geq 1,\;n=\left|N\left[i\right]\right|$$

According to Glover et al. [32], for n=1, the constraint is given by $P\cdot {\left(X_{1}-1\right)}^{2}$, where *P* is the penalty coefficient. For n=2, the constraint becomes $P\cdot (1-X_{1}-X_{2}-X_{1}\cdot X_{2})$. For n≥3, the inequality constraint must be converted into an equality constraint by introducing slack variables, as shown in Equation (7).

(7) $$X_{1}+X_{2}+\cdots +X_{n}-S-1=0$$

It is clear that *S* lies within the range [0,n−1]. We now express *S* as a combination of binary (0–1) variables. Since *S* can take any integer value within [0,n−1], it can be represented as follows:

(8) $$S=\sum_{i=1}^{bl_{n-1}-1}X_{i}^{\prime }\cdot 2^{i-1}+\left(n-1-\sum_{i=1}^{bl_{n-1}-1}2^{i-1}\right)\cdot X_{bl_{n-1}}^{\prime }$$

The variable $X_{*}^{\prime }\in \left\{0,1\right\}$ is an additional binary variable introduced to represent slack variables, and $bl_{n}$ denotes the binary length of *n*. By substituting the expression from Equation (8) into Equation (7) and squaring the entire equation, we obtain the quadratic penalty. For Constraint (4), it can be represented as $P\cdot (X_{i}\cdot X_{j})$. The QUBO model for the IDP is presented in Equation (9). In Equation (9), the vertices in *V* are partitioned based on |N[i]|=1,2, and |N[i]|≥3, which allows for the separate treatment of Constraint (2).

(9) $$\begin{array}{c} \min_{\left\{X,X^{\prime }\right\}}\;\sum_{i=1}^{\left|V\right|}X_{i}\\ +\sum_{i\in V,|N[i]|\geq 3}P\cdot [\sum_{j\in N[i]}X_{j}-(\sum_{i=1}^{bl_{|N[i]|-1}-1}X_{i}^{\prime }\cdot 2^{i-1}+\left(|N\left[i\right]\right|-1-\sum_{i=1}^{bl_{|N[i]|-1}-1}2^{i-1})\cdot X_{bl_{|N[i]|-1}}^{\prime }{)-1]}^{2}\\ +\sum_{i\in V,|N[i]|=2,N[i]=\{j,k\}}P\cdot \left(1-X_{j}-X_{k}-X_{j}\cdot X_{k}\right)\\ +\sum_{i\in V,|N[i]|=1,N[i]=\{j\}}P\cdot {\left(X_{j}-1\right)}^{2}\\ +\sum_{ij\in E}P\cdot X_{i}\cdot X_{j} \end{array}$$

### 2.3. QUBO Model → Hamiltonian

To convert the objective function in Equation (9) into a Hamiltonian, we substitute the binary variables $X_{i}$ with new variables $s_{i}\in \left\{-1,1\right\}$. The substitution process is as follows:

(10) $$X_{i}=\frac{s_{i}+1}{2}$$

Then, we have

(11) $$\begin{array}{c} \min_{\left\{s,s^{\prime }\right\}}\;\sum_{i=1}^{\left|V\right|}\frac{s_{i}+1}{2}\\ +\sum_{i\in V,|N[i]|\geq 3}P\cdot [\sum_{j\in N[i]}\frac{s_{j}+1}{2}\\ \;-(\sum_{i=1}^{bl_{|N[i]|-1}-1}\frac{s_{i}^{\prime }+1}{2}\cdot 2^{i-1}+\left(|N\left[i\right]\right|-1-\sum_{i=1}^{bl_{|N[i]|-1}-1}2^{i-1})\cdot \frac{s_{bl_{|N[i]|-1}}^{\prime }+1}{2}{)-1]}^{2}\\ +\sum_{i\in V,|N[i]|=2,N[i]=\{j,k\}}P\cdot \left(1-\frac{s_{j}+1}{2}-\frac{s_{k}+1}{2}-\frac{s_{j}+1}{2}\cdot \frac{s_{k}+1}{2}\right)\\ +\sum_{i\in V,|N[i]|=1,N[i]=\{j\}}P\cdot {\left(\frac{s_{j}+1}{2}-1\right)}^{2}\\ +\sum_{ij\in E}P\cdot \frac{s_{i}+1}{2}\cdot \frac{s_{j}+1}{2} \end{array}$$

By replacing *s* and $s^{\prime }$ with the Pauli-Z operator $\sigma^{z}$, we ultimately obtain the Hamiltonian.

(12) $$\begin{array}{c} H_{c}=\sum_{i=1}^{\left|V\right|}\frac{\sigma_{i}^{z}+1}{2}\\ +\sum_{i\in V,|N[i]|\geq 3}P\cdot [\sum_{j\in N[i]}\frac{\sigma_{j}^{z}+1}{2}\\ \;-(\sum_{i=1}^{bl_{|N[i]|-1}-1}\frac{{\left(\sigma_{i}^{z}\right)}^{\prime }+1}{2}\cdot 2^{i-1}+\left(|N\left[i\right]\right|-1-\sum_{i=1}^{bl_{|N[i]|-1}-1}2^{i-1})\cdot \frac{{\left(\sigma_{bl_{|N[i]|-1}}^{z}\right)}^{\prime }+1}{2}{)-1]}^{2}\\ +\sum_{i\in V,|N[i]|=2,N[i]=\{j,k\}}P\cdot \left(1-\frac{\sigma_{j}^{z}+1}{2}-\frac{\sigma_{k}^{z}+1}{2}-\frac{\sigma_{j}^{z}+1}{2}\cdot \frac{\sigma_{k}^{z}+1}{2}\right)\\ +\sum_{i\in V,|N[i]|=1,N[i]=\{j\}}P\cdot {\left(\frac{\sigma_{j}^{z}+1}{2}-1\right)}^{2}\\ +\sum_{ij\in E}P\cdot \frac{\sigma_{i}^{z}+1}{2}\cdot \frac{\sigma_{j}^{z}+1}{2} \end{array}$$

## 3. QAOA

In this section, we begin by introducing the fundamental concepts of the QAOA. For a quantum system comprising *n* qubits, |z〉 denotes a state vector in the $2^{n}$-dimensional Hilbert space, where the bit string *z* is expressed as $z=z_{0}z_{1}z_{2}\cdots z_{n-1}$. The QAOA starts by applying the Hadamard gate to prepare the initial state |s〉 from the state $|\underset{n}{\underset{︸}{00\ldots 0}}\rangle$.

(13) $$|s\rangle =\underset{n}{\underset{︸}{\hat{H}⊗\hat{H}\cdots ⊗\hat{H}}}|\underset{n}{\underset{︸}{00\cdots 0}}\rangle =\frac{1}{\sqrt{2^{n}}}\cdot \sum_{z}|z\rangle$$

Then, we use two parametric unitary transformations, U(C,γ) and U(B,β),

$$\begin{array}{cc} U(C,\gamma ) & =e^{-i\gamma H_{c}}\\ U(B,\beta ) & =e^{-i\beta B} \end{array}$$

where $C=H_{c}$, $B=\sum_{j=1}^{n}\sigma_{j}^{x}$, γ∈[0,2π], and β∈[0,π]. By applying these two types of unitary transformations to the initial state |s〉 and repeating the process *q* times, we arrive at the final state |γ,β〉, as described in Equation (14). In this paper, *q* is also referred to as the layer number of the QAOA.

(14) $$|\gamma ,\beta \rangle =U\left(B,\beta_{q}\right)U\left(C,\gamma_{q}\right)\cdots U\left(B,\beta_{1}\right)U\left(C,\gamma_{1}\right)|s\rangle$$

After obtaining |γ,β〉, we can measure the expectation value of $H_{c}$.

(15) $$F_{q}\left(\gamma ,\beta \right)=\langle \gamma ,\beta |H_{c}|\gamma ,\beta \rangle$$

**Figure 1:**
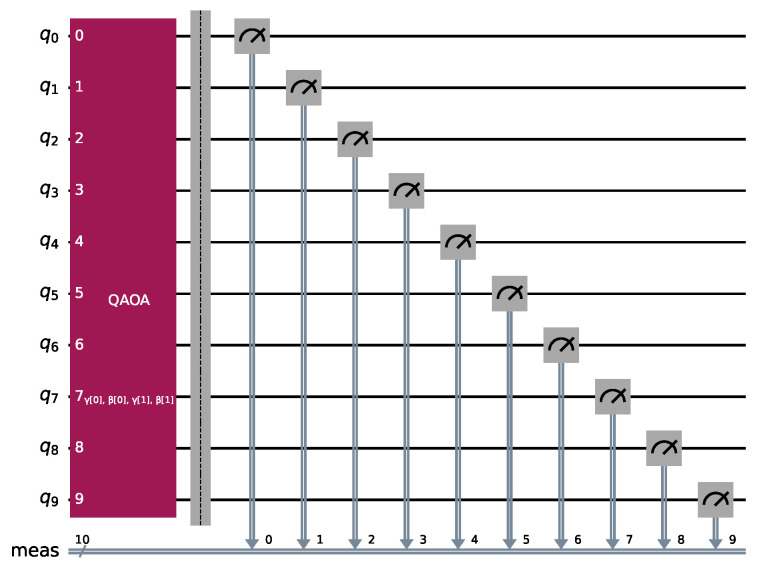
Basic working flow of QAOA with 2 layers and 10 qubits.

As a reference, Figure 1 shows the quantum circuit for 10 qubits with two layers.

For the IDP in this paper, our task is to find such γ and β with the minimal expectation value of $H_{c}$. The QAOA is a hybrid algorithm that combines a classical optimizer with a quantum computer. We can use a classical optimizer to search for such γ and β. Next, we summarize the steps of using the QAOA to solve the IDP:

Step 1: Convert the IDP into a QUBO model.
Step 2: Transform the QUBO model into a Hamiltonian.
Step 3: Set the layer number *q* and convert the Hamiltonian into a quantum circuit. IBM’s Qiskit integrates many tools, such as QAOAAnsatz. We can generate the quantum circuit by inputting the Hamiltonian and the layer number.
Step 4: Initialize γ and β for each layer.
Step 5: Use a classical optimizer, such as COBYLA, to optimize γ and β. The optimization process terminates when the maximum number of iterations is reached or the predefined function tolerance is satisfied, yielding $\gamma_{*}$ and $\beta_{*}$.
Step 6: Update the quantum circuit with $\gamma_{*}$ and $\beta_{*}$.
Step 7: Perform multiple samplings on the updated quantum circuit and output the bit string $z_{*}$ with the highest probability.
Step 8: Derive the IDS of the graph based on $z_{*}$.

## 4. Experiment

**Figure 2:**
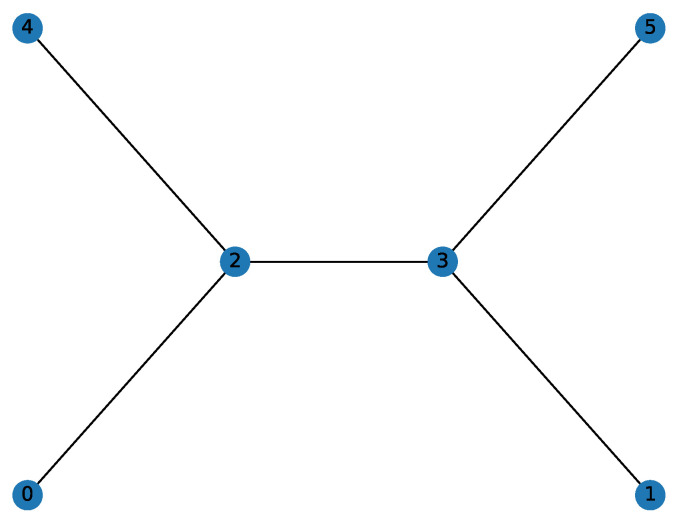
An unweighted graph with 6 nodes and 5 edges.

In this section, we evaluate the performance of the QAOA-based algorithm in solving the IDP. The graph used for this analysis is shown in Figure 2. This unweighted graph consists of 6 vertices, with the optimal DS and IDS being distinct. Specifically, the optimal DS is {2,3}, while the optimal IDS has two possible solutions due to the graph’s symmetry: {0,4,3} or {1,5,2}. Selecting a graph where the optimal DS and IDS differ is crucial for assessing the QAOA’s ability to solve the IDP rather than the DP. Additionally, the graph’s symmetry leads to multiple potential optimal IDSs, which should be reflected in the final probability distribution. Ideally, the two bit strings with the highest probabilities should correspond to the IDSs {0,4,3} and {1,5,2}, with probabilities that are either equal or very close.

Based on the modeling method outlined in Section 2, the QUBO formulation of the IDP for this graph is given in Equation (16), which requires 10 qubits. Each term in Equation (16) is explained in Table 1. The corresponding Hamiltonian for this model can be derived by replacing $x_{*}$ with $\frac{s_{*}+1}{2}$ and substituting $s_{*}$ with $\sigma_{*}^{z}$. For brevity, the detailed steps of this process are omitted here. Notably, the 10 decision variables in Equation (16) correspond to the 10 qubits in the quantum system. When measuring the energy of this 10-qubit system, the corresponding bit string *z* will have a length of 10, such as $z=z_{0}z_{1}\cdots z_{5}\cdots z_{9}$. However, as the optimization model introduces slack variables, we focus primarily on the values of $z_{0},z_{1},\cdots ,z_{5}$, as these determine which vertices belong to the IDS. For example, when the bit string $z_{0}z_{1}z_{2}z_{3}z_{4}z_{5}=100110$, it indicates that the set of vertices {0,3,4} forms an IDS. Thus, when calculating the probability distribution of bit strings, we aggregate the probabilities of all bit strings with identical values for the first 6 qubits. For instance, if the sampling probability is 0.01 for z=0000000100 and 0.02 for z=0000001100, the combined sampling probability for z=000000 would be 0.03. In subsequent sections, when referring to a bit string, we will use the merged 6-bit string representing the first 6 qubits.

(16) $$\begin{array}{cc} minimize\; & x_{0}+x_{1}+x_{2}+x_{3}+x_{4}+x_{5}\\ \; & +P\cdot \left(1-x_{0}-x_{2}+x_{0}x_{2}\right)\\ \; & +P\cdot \left(1-x_{1}-x_{3}+x_{1}x_{3}\right)\\ \; & +P\cdot {\left(x_{2}+x_{0}+x_{4}+x_{3}-\left(x_{6}+2x_{7}\right)-1\right)}^{2}\\ \; & +P\cdot {\left(x_{3}+x_{1}+x_{2}+x_{5}-\left(x_{8}+2x_{9}\right)-1\right)}^{2}\\ \; & +P\cdot \left(1-x_{4}-x_{2}+x_{4}x_{2}\right)\\ \; & +P\cdot \left(1-x_{5}-x_{3}+x_{5}x_{3}\right)\\ \; & +P\cdot x_{0}x_{2}+P\cdot x_{1}x_{3}+P\cdot x_{2}x_{4}+P\cdot x_{2}x_{3}+P\cdot x_{3}x_{5} \end{array}$$

In this experiment, we used IBM’s qasm_simulator as the backend, with QAOAAnsatz serving as the quantum circuit generator and BaseSamplerV1 as the sampler. The optimization method employed was COBYLA, with a default function tolerance of $10^{-8}$. The cost function used for optimization was the Conditional Value-at-Risk (CVaR), which has been widely adopted in various studies to improve both the convergence probability and speed when applied to QAOA [33,34]. As described by Kolotouros and Wallden [33], a variant of CVaR called ascending-CVaR is defined in Equation (17). Here, *K* represents the total number of samples, and the energy values obtained from the *K* measurements are sorted in ascending order. $H_{c,i}$ denotes the energy value of the *i*-th measurement. The cost function is then calculated as the average of the first ⌈alpha·K⌉ energy values. This approach prioritizes lower-energy regions, which are more closely related to the optimal solution, rather than considering the entire energy distribution, as in Equation (15), thereby accelerating the convergence process.

(17) $$CVaR_{alpha}=\frac{1}{\left\lceil alpha\cdot K\right\rceil }\cdot \sum_{i=0}^{\left\lceil alpha\cdot K\right\rceil }H_{c,i}$$

COBYLA is a well-known gradient-free optimization algorithm. In each iteration, COBYLA constructs a trust region based on the current CVaR value, using the parameters γ and β. It then selects a set of sample points within this trust region to build a linear approximation model. By solving this linear model, the next iteration point is determined. This process is repeated until the optimal solution is approached. The optimization terminates when the change in the CVaR value is smaller than the function tolerance or when the maximum number of iterations is reached. According to the cost function definition in Equation (17), the convergence of COBYLA is achieved when the change in the average of the first ⌈alpha·K⌉ energy measurements becomes smaller than the function tolerance. For the initial values of γ and β, we adopted the initialization method proposed by Sack and Serbyn [35]. The key parameters considered in the experiment include the number of layers *q* in the QAOA, the alpha parameter of the CVaR, the penalty coefficient *P* in the QUBO model, and the maximum number of iterations allowed for COBYLA.

The experiment is divided into two main phases: fundamental testing and robustness testing. In the fundamental testing phase, we assess the performance of the QAOA in solving the IDP using specific parameter settings. The robustness testing phase focuses on comparing the computational results of the QAOA across different parameter configurations. Additionally, this section includes an analysis of the complexity of the QAOA.

### 4.1. Fundamental Testing

**Figure 3:**
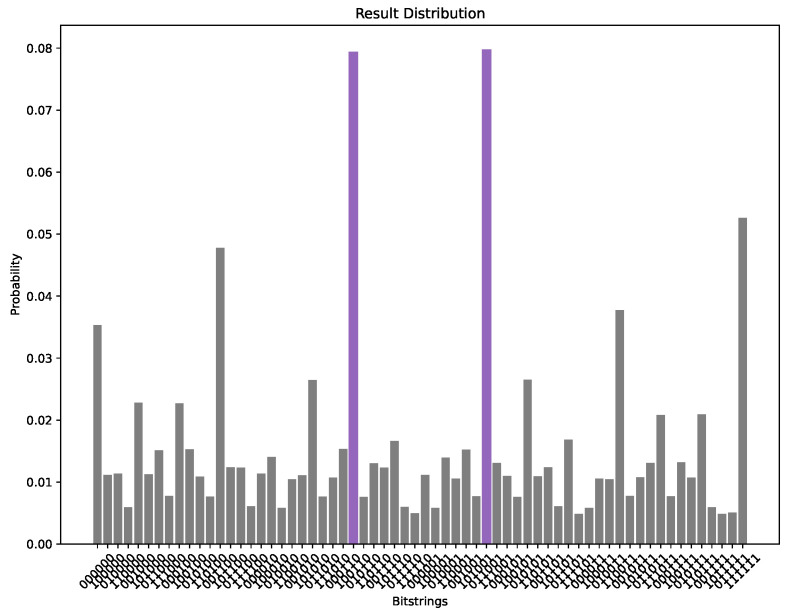
The probability distribution of the bit strings when q=15, alpha=0.3, P=4.5, and maximum iterations = 10,000. The sampling probabilities for the two most probable bit strings are highlighted in purple.

**Figure 4:**
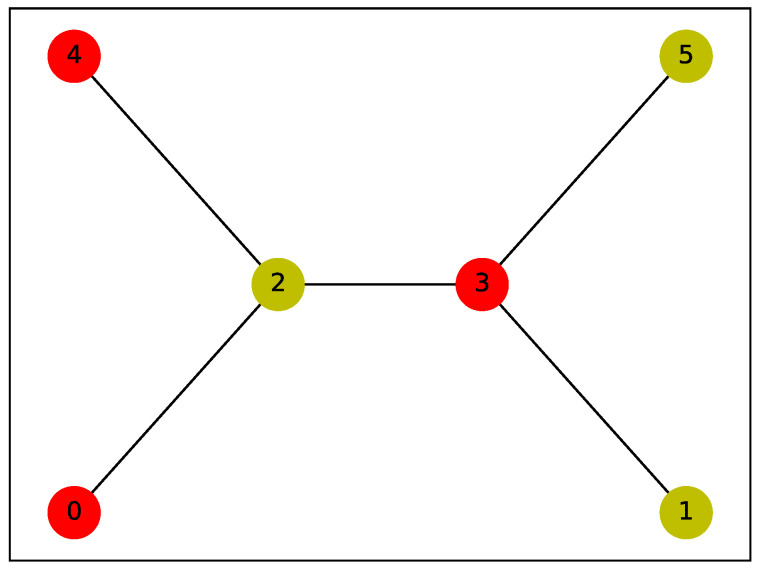
Visualization of the IDS {1,2,5}. The yellow vertices represent the elements of the IDS, while the red vertices indicate the dominated vertices.

**Figure 5:**
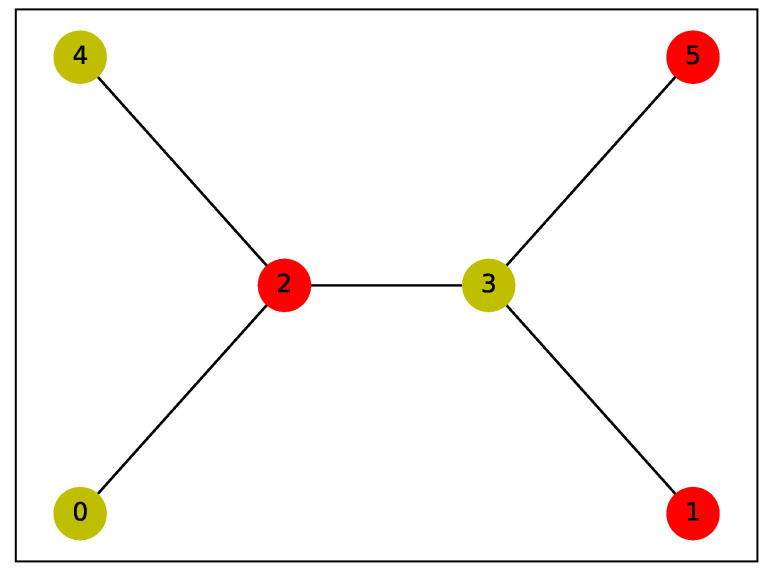
Visualization of the IDS {0,3,4}. The yellow vertices represent the elements of the IDS, while the red vertices indicate the dominated vertices.

The parameters were set to q=15, alpha=0.3, and P=4.5, where *P* is 0.75 times the number of nodes in the selected graph, with a maximum of 10,000 iterations. Under these conditions, we employed the QAOA to solve the IDP for the 6-node graph shown in Figure 2. Figure 3 presents the probability distribution of the final sampling results, where the bit strings z=011001 and z=100110 have probabilities of 0.0797 and 0.0793, respectively, significantly higher than those of other configurations. Visualizing these results on the graph, we observe that the outcome is a consequence of the inherent symmetry of the graph (as shown in Figure 4 and Figure 5), demonstrating the effectiveness of the QAOA in solving the IDP. Although the sampling probabilities of the two optimal bit strings are higher than those of other configurations, both remain on the order of 0.01. Given reasonable choices for the penalty coefficients, this may be due to two potential causes. First, a low number of layers might have been used, as suggested by Farhi et al. [17]. Second, the classical optimizer may have become trapped in a local minimum. Since COBYLA is a gradient-free optimization method, it may have disadvantages in escaping local minima compared to gradient-based algorithms like BFGS. Developing a suitable gradient and integrating gradient-based methods, such as BFGS, with the QAOA represents a promising direction for future research.

**Figure 6:**
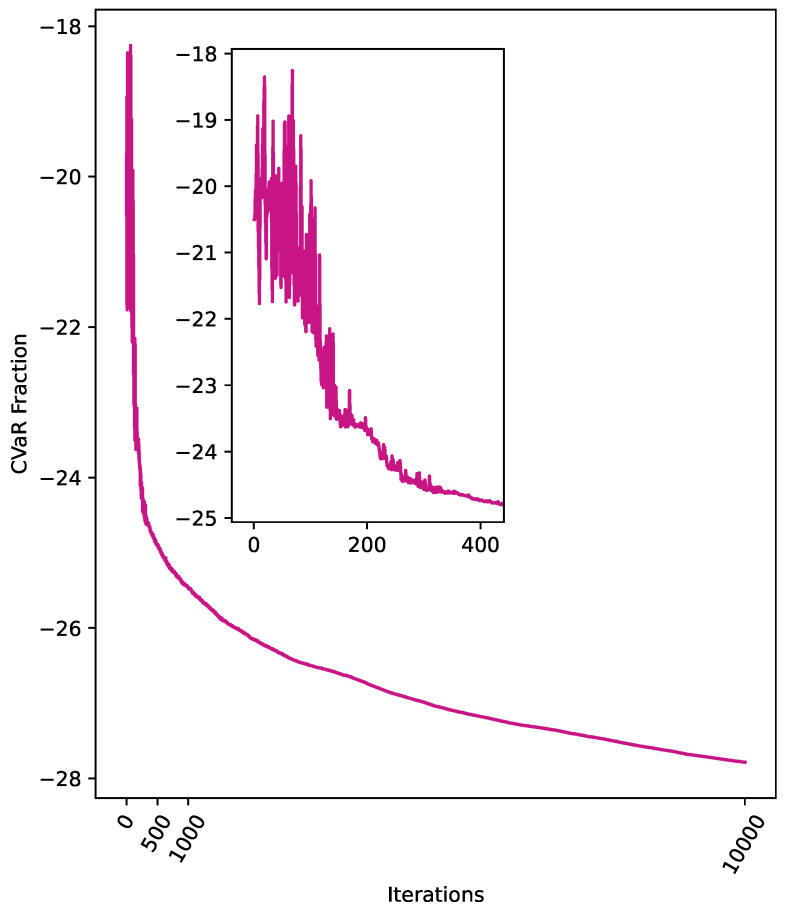
The cost of the QAOA for q=15, alpha=0.3, P=4.5, and a maximum of 10,000 iterations. The zoomed-in region focuses on iterations within the range [0,400].

In Figure 6, we track the cost evolution over the course of the iterations. A sharp decrease in cost is observed within the first [0,200] iterations, followed by a more gradual decline thereafter. One possible explanation for this behavior is that the penalty term initially outweighs the original objective function. The significant reduction in cost during the [0,200] interval may be attributed to the optimizer initially focusing on bit strings *z* that drive the penalty terms in the objective function to zero. For example, in the case of the largest IDS of the graph, {0,1,4,5}, the corresponding bit string is z=110011. Once the penalty terms are minimized, the optimization shifts toward finding bit strings that minimize the size of the IDS. Although the cost decreases more gradually after 200 iterations, it continues to show a clear downward trend. However, this gradual decline does not alter the fact that z=011001 and z=100110 remain the two bit strings with the highest probabilities. Further exploration may enhance the probabilities of obtaining correct and optimal results. At the current parameter settings, the probabilities for these bit strings are 0.197 and 0.159, respectively, as shown in Table 2. The calculation of correct and optimal probabilities is as follows: After determining $\gamma_{*}$ and $\beta_{*}$, multiple samplings are performed. The correct probability is the sum of the sampling probabilities of the bit strings that satisfy the IDS condition. The optimal probability is the sum of the sampling probabilities of the bit strings whose corresponding vertex sets satisfy the IDS condition and are of optimal size.

### 4.2. Robustness Testing

In this section, we perform a robustness analysis on the four key parameters of the QAOA-based algorithm: alpha, *q*, *P*, and the maximum number of iterations. A total of 144 parameter combinations were tested, varying q∈{10,15,20}, alpha∈{0.3,0.5,0.7}, P∈{3,4.5,6,9}, and the maximum number of iterations ∈{100,500,1000,10,000}. The possible values of *P* correspond to 0.5, 0.75, 1, and 1.5 times |V|, where |V| represents the upper bound of |IDS|. This range is inspired by the work of Glover et al. [32], who recommend setting *P* between 0.75 and 1.5 times the original objective function. In addition to these values, we have also included 0.5·|V| as a further option. Out of the tested parameter combinations, 97 produced a $z_{*}$ that satisfied the IDS condition, with 22 of these combinations yielding the optimal IDS. This observation indicates that the effectiveness of the QAOA in solving the IDP is highly dependent on the choice of parameters. By analyzing the performance of the QAOA under different parameter settings, we can better inform the parameter tuning process for future research on using the QAOA to solve the IDP.

**Figure 7:**
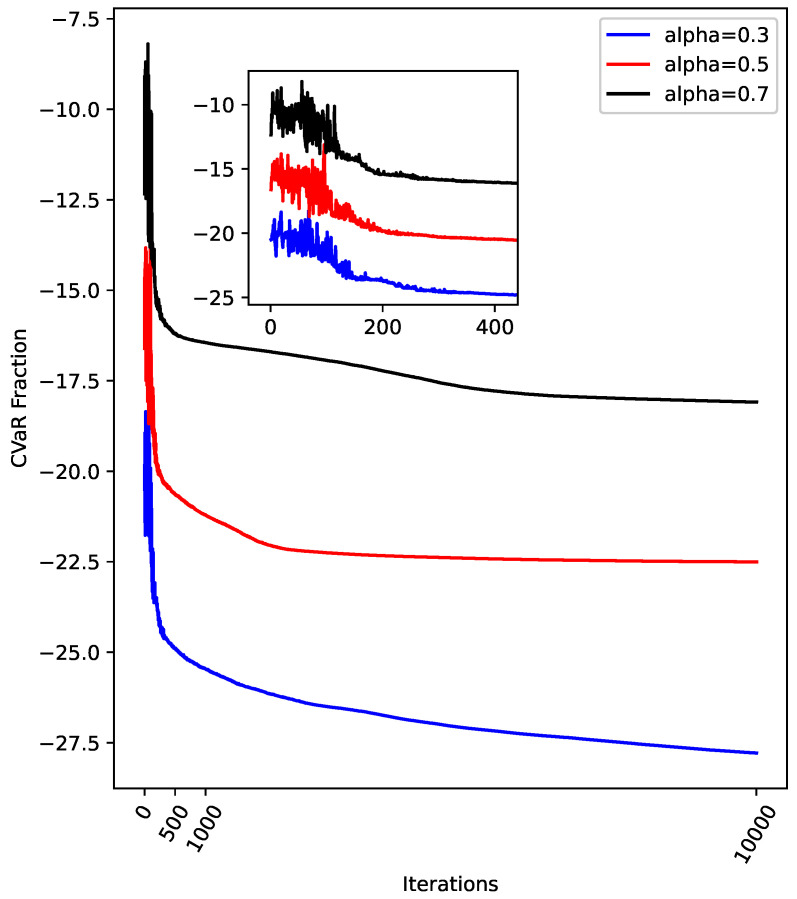
A comparison of the cost for alpha=0.3, 0.5, and 0.7. The values of *q*, *P*, and the maximum number of iterations are set to 15, 4.5, and 10,000, respectively. The zoomed-in region focuses on iterations within the range [0,400].

**Figure 8:**
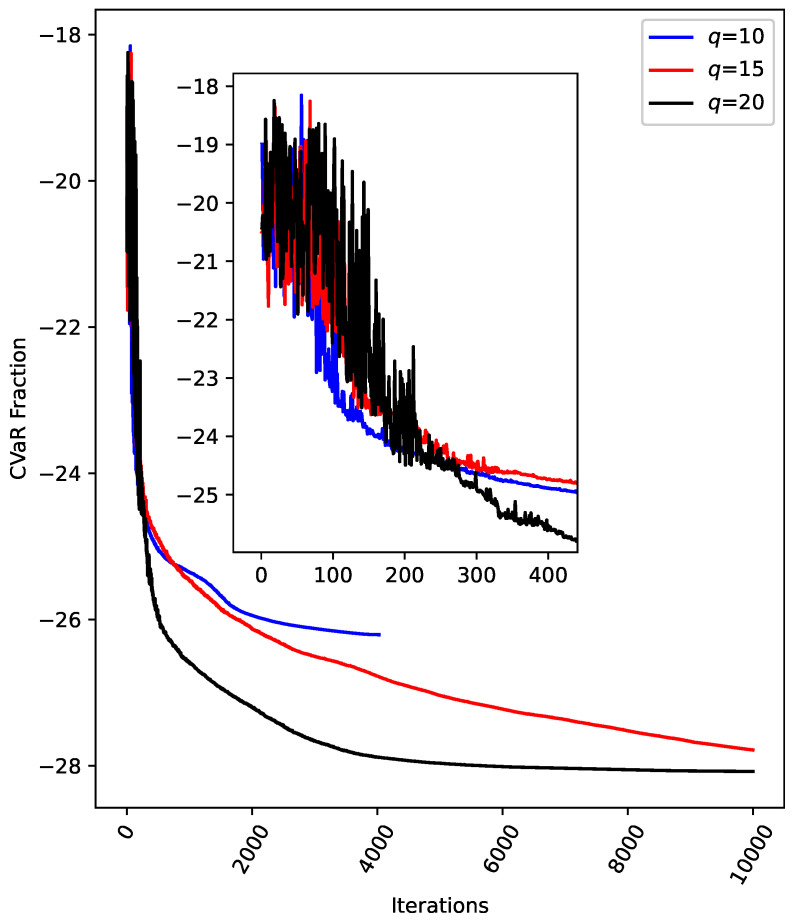
A comparison of the cost for q=10, 15, and 20. The values of *p*, alpha, and the maximum number of iterations are set to 4.5, 0.3, and 10,000, respectively. The zoomed-in region focuses on iterations within the range [0,400].

In Figure 7, we set q=15, P=4.5, and a maximum of 10,000 iterations and compare the changes in cost for alpha=0.3, 0.5, and 0.7. As observed in Figure 6, the cost decreases sharply within the first [0,200] iterations for all values of alpha. Beyond this range, the reduction in cost becomes more gradual. Overall, the cost reduction trend is consistent across the different values of alpha. The data in Figure 7 indicate that the QAOA achieves good convergence when solving the IDP, regardless of the alpha setting. In Figure 8, we compare the cost convergence for q=10,15,20, with P=4.5, alpha=4.5, and a maximum of 10,000 iterations. It is evident that when q=10, the cost converges before reaching the maximum number of iterations, meeting the function tolerance. For q=15 and q=20, while the cost quickly levels off for q=20, the cost for q=15 continues to decrease significantly, even at 10,000 iterations. This suggests that, had the maximum number of iterations been further increased, the cost for q=15 would likely continue to decrease. From the perspective of the QAOA, as the number of layers increases, $F_{q}\left(\gamma ,\beta \right)$ tends to approach the optimal objective function value [17]. However, for COBYLA, a larger number of layers may negatively affect convergence speed and increase the likelihood of getting trapped in a local minimum. Referring to Figure 8, while the cost converges the fastest when q=10, the CVaR value at convergence is the largest, likely due to the limitation imposed by the smaller number of layers. For q=20, despite having the smallest CVaR value within the set range of maximum iterations, it is possible that the optimization prematurely converges to a local minimum. Therefore, when applying the QAOA to solve the IDP, careful consideration must be given to the choice of the number of layers. A basic recommendation is that when fast convergence is prioritized, starting with a small-layer QAOA structure is advisable. However, when seeking higher-quality solutions, a slightly larger-layer QAOA structure is necessary. In the case of larger-layer QAOA structures, caution should be exercised regarding the potential for getting trapped in a local minimum.

**Figure 9:**
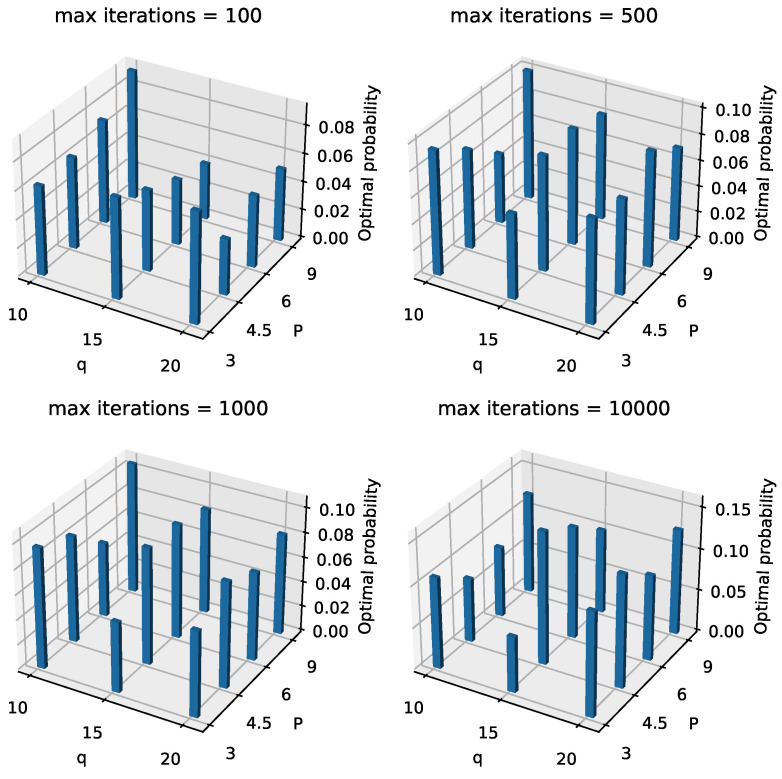
Landscape of optimal probabilities of different *q*, *P*, and maximum iterations when alpha = 0.3.

**Figure 10:**
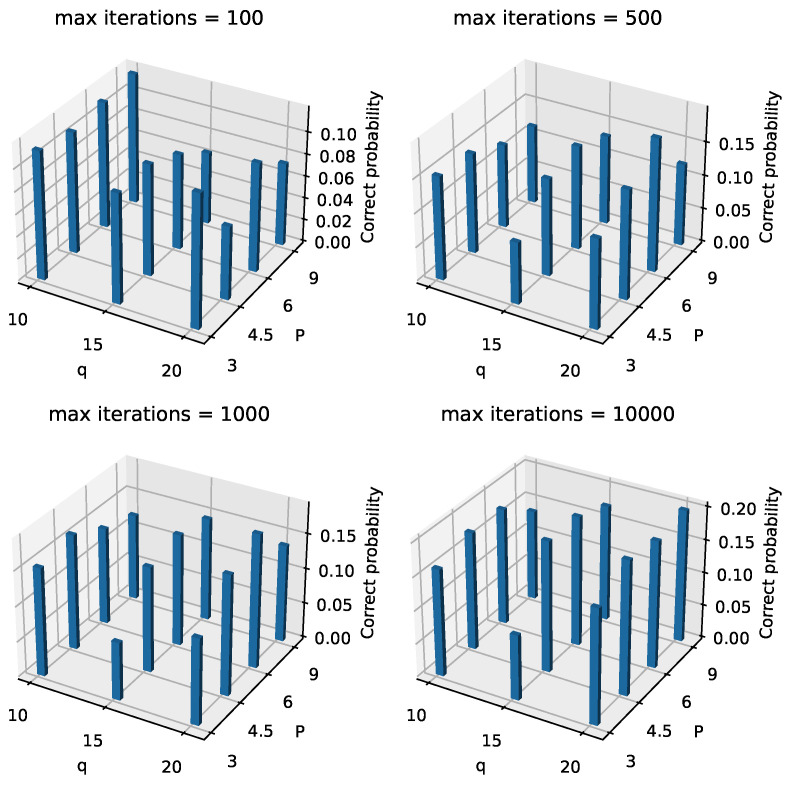
Landscape of correct probabilities of different *q*, *P*, and maximum iterations when alpha = 0.3.

Next, in Figure 9 and Figure 10, we present the optimal and correct probabilities for different combinations of *q*, *P*, and maximum iterations, with alpha=0.3. On a macro level, both the optimal and correct probabilities generally increase as the number of maximum iterations increases. When the maximum iteration number is set to 100, many parameter combinations result in low probabilities for both categories, but these probabilities improve significantly as the number of iterations increases. For instance, Table 3 shows how these probabilities change as the number of iterations increases, with q=15, alpha=0.3, and P=4.5. This behavior aligns with our expectations. Additionally, based on observations from Figure 9 and Figure 10, we note that when the maximum iteration number is small, the small-layer QAOA structure outperforms the large-layer QAOA structure in terms of both types of probabilities. This finding contradicts the theoretical prediction that the expectation of $H_{c}$ should approach the optimal value as the number of layers increases. Upon further analysis, we hypothesize that this behavior arises because the larger-layer QAOA requires optimizing a greater number of angles compared to the smaller-layer QAOA, as the number of angles is twice the number of layers. For classical optimization algorithms, as the number of angles increases, if the number of iterations is insufficient, the classical optimizer becomes a bottleneck in the process. As the number of iterations increases, the large-layer QAOA structure gradually begins to outperform the small-layer structure in terms of both types of probabilities. Although the large-layer QAOA does not always outperform the small-layer structure for all parameter combinations (for example, in Figure 9, where maximumiterations=1000, q=15, and P=3), the robustness of the tests allows us to observe this trend in most other combinations. From the analysis of Figure 9 and Figure 10, we draw the following insights: when aiming to solve the problem with a smaller maximum number of iterations, a smaller-layer QAOA structure can be used. However, when seeking to maximize the probability of obtaining an IDS or optimal IDS, a larger-layer QAOA structure should be prioritized.

In Table 4, we compare the impact of different penalty coefficients on the correct and optimal probabilities. The four values of *P* correspond to 0.5, 0.75, 1, and 1.5 times the number of vertices, which serves as an upper bound for the size of the IDS. These values reflect varying proportions between the penalty term and the original objective function. Our analysis reveals no clear correlation between *P* and the two probability metrics. Furthermore, even for two closely related penalty coefficients, such as P=3 and P=4.5, we observe significant differences in their corresponding correct and optimal probabilities. This highlights the importance of carefully selecting *P*, as emphasized by Glover et al. [32]. According to Glover et al. [32], it is recommended to set *P* to between 0.75 and 1.5 times the original objective function, which aligns with our experimental findings. Additionally, in Figure 9 and Figure 10, when the number of maximum iterations is sufficiently large, both types of probabilities show no significant shortcomings within the weight range recommended by Glover et al. [32]. This further supports the reasonableness of the chosen range for the penalty coefficient. However, it is important to note that we have not conducted extensive testing outside the range recommended by Glover et al. [32]. Therefore, the results related to the penalty coefficient in this paper serve primarily as a validation of the recommendations made by Glover et al. [32].

**Figure 11:**
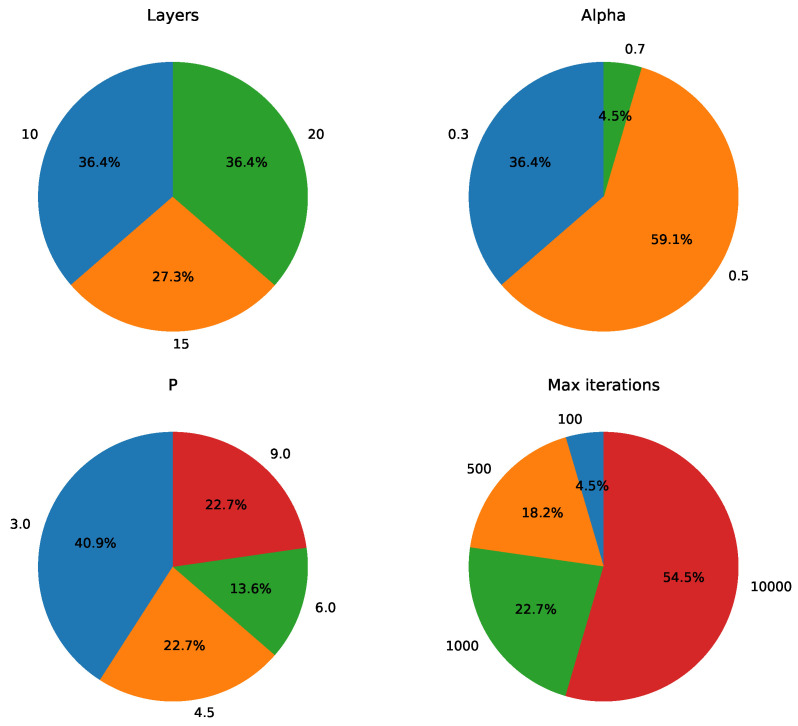
Parameter distribution of experiments in which $z_{*}$ is an optimal IDS.

**Figure 12:**
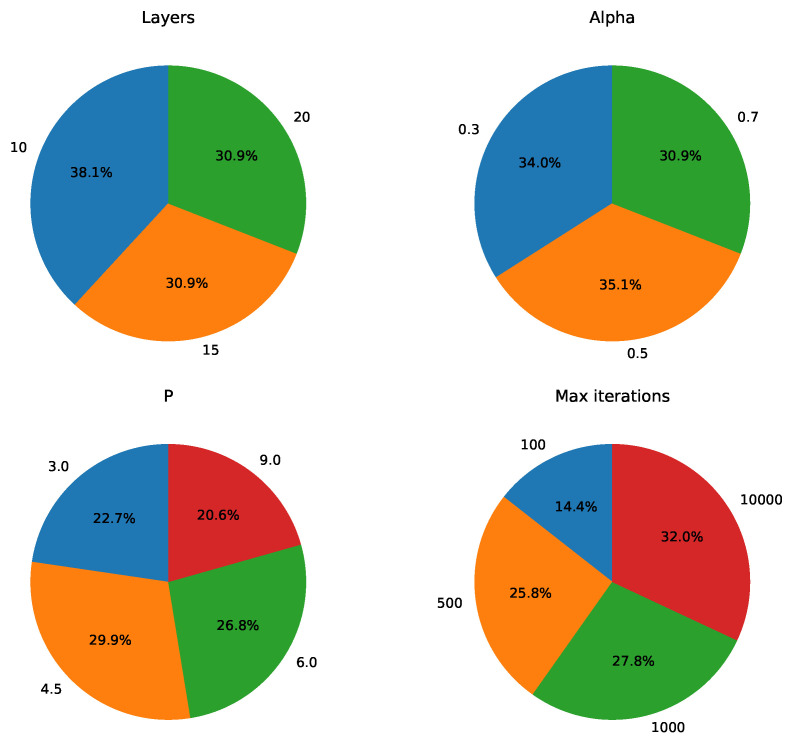
Parameter distribution of experiments in which $z_{*}$ is an IDS.

As part of the further analysis of the parameter dependency of the QAOA, we recorded the parameter distribution results for all experiments where $z_{*}$ corresponds to the optimal IDS or an IDS. Taking the number of layers *q* as an example, in the 144 experiments, $z_{*}$ was the IDS in 97 of them. Suppose that in these 97 experiments, the number of experiments with q=10, q=15, and q=20 is $n_{1}$, $n_{2}$, and $n_{3}$, respectively. The corresponding ratios are $\frac{n_{1}}{97}$, $\frac{n_{2}}{97}$, and $\frac{n_{3}}{97}$. Similarly, for alpha, *P*, and maximum iterations, we compute the ratios in the same manner and present the results in pie charts. The statistical results for $z_{*}$ being the optimal IDS or an IDS are shown in Figure 11 and Figure 12.

Before analyzing the results, it is important to note that the focus of this statistical analysis differs from that of Figure 9 and Figure 10. For example, when $z_{*}$ is the optimal IDS, the corresponding bit string has the highest sampling probability globally, but this advantage may be small. In other words, the sampling probability of the optimal IDS may not be large, as long as it is slightly higher than that of other bit strings. In contrast, Figure 9 reports the sampling probabilities for the bit string corresponding to the optimal IDS. There may be cases where the optimal probability is large, but $z_{*}$ is not the optimal IDS. When using QAOA in practice, we can directly consider $z_{*}$ as the final result, in which case we are primarily concerned with whether $z_{*}$ is the optimal IDS. Alternatively, we may filter the sampling results and focus more on the size of the optimal probability.

After clarifying the distinction between these two types of metrics, we now turn to the analysis of Figure 11 and Figure 12. We observe that for both $z_{*}$ being the optimal IDS and an IDS, there is a clear dependency on the maximum number of iterations, while no significant trend is observed for the number of layers. While the small-layer QAOA may be constrained by theoretical performance limitations, it offers the advantage of better convergence. As a result, the small-layer QAOA can still produce an optimal PDS, although the probability of obtaining the optimal PDS may not be very high. This implies that if our goal is to obtain optimal results from the QAOA without focusing on maximizing the optimal probability, we need not excessively increase the number of layers. Instead, we should focus on using a larger maximum number of iterations. Regarding the penalty coefficient *P*, the results in Figure 11 and Figure 12 suggest a potential direction for further exploration. Specifically, *P* values smaller than the range recommended by Glover et al. [32] (e.g., P=0.5·|V|) might still yield good results. Based on our observations of Equation (16), we find that the penalty term contains 11 terms, and excessively large values of *P* could cause a large discrepancy between the penalty term and the original objective function, potentially affecting the convergence of the cost. According to the statistical results for *P* in Figure 11, we hypothesize that slightly reducing *P* from its current setting might still yield good results. For alpha, Figure 11 shows that alpha=0.5 occupies a significantly higher proportion, while no such trend is observed in Figure 12. Given the small number of experiments where $z_{*}$ is the optimal IDS compared to those where $z_{*}$ is the IDS, we cannot conclusively determine whether the observed difference is due to statistical fluctuations. Since $z_{*}$ being the optimal IDS implies that $z_{*}$ is first an IDS, and the statistical sample for the optimal IDS is relatively small, we are inclined to accept the statistical results for the IDS. Based on these considerations, we believe further testing is needed to better understand the dependency of $z_{*}$ being the optimal IDS on alpha.

### 4.3. Complexity Analysis

Zhang et al. [20] analyzed the time complexity of the QAOA, which consists of two components. The first component, O[poly(q)], represents the time complexity associated with the multi-layer structure of the QAOA, which is the most time-consuming aspect. The second component pertains to the time complexity of the optimization algorithm used. In this paper, we employ COBYLA as the optimization algorithm, which has a time complexity of O[m], where *m* represents the maximum number of iterations. Therefore, the overall time complexity of the QAOA can be expressed as O[poly(q)+poly(m)].

In Table 5, we compare the time complexity of the QAOA with other algorithms previously used to solve the IDP. Our analysis shows that the QAOA offers a significant advantage in terms of time complexity when solving the IDP and can be executed on a quantum computer. However, this does not imply that the QAOA is superior in all aspects for solving the IDP. Based on the experimental results and analyses presented in Section 4.1 and Section 4.2, we conclude that the QAOA is effective for solving the IDP, provided that the algorithm’s parameters are carefully selected. Additionally, there is still room for improvement in both the accuracy of the results and the probability of obtaining the optimal solution.

Additionally, when executing the QAOA on classical computers, it is important to consider memory constraints. This requirement arises because, during the sampling process for a combinatorial optimization problem involving *n* qubits, a matrix of size $2^{n}$ by the number of samples per bit string must be stored locally. As a result, running the QAOA on a local quantum simulator requires substantial memory overhead.

## 5. Conclusions

In this paper, we explored the use of the quantum algorithm QAOA to solve the IDP. We first modeled the IDP as a 0-1 integer programming problem and converted various constraints into corresponding quadratic penalties, which were added to the original objective function, resulting in the QUBO formulation of the IDP. This QUBO model was then transformed into a Hamiltonian, completing the preparation for inputting the IDP into the quantum algorithm. We applied the QAOA to solve the IDP, a hybrid approach that combines quantum computation with classical optimization. By adjusting the γ and β parameters for each layer of the QAOA through classical optimization, we gradually reduced the expectation value of $H_{c}$, which corresponds to the objective function of the IDP.

In the experimental section, we conducted both fundamental and robustness testing. In fundamental testing, we examined the probability distribution and cost variation results of the QAOA in solving the IDP with specific parameters. The results showed that the QAOA-based algorithm is effective for solving the IDP, as the final sampling outcomes reflected multiple IDSs due to the inherent symmetry of the graph. Additionally, the cost gradually flattened after sharp fluctuations within a narrow range, indicating the good convergence performance of the QAOA. In robustness testing, we made the following key observations: (1) By comparing the cost variation curves for different values of *alpha* and *q*, we found that the CVaR-based cost function showed no significant dependency on *alpha* in terms of convergence speed but did exhibit a tendency with respect to the number of layers. This test also suggested that large-layer QAOA structures may be more prone to getting trapped in local minima. (2) For both optimal and correct probabilities, we compared and analyzed their behavior under different parameters. The results indicated that both types of probabilities showed a slight advantage with larger layer numbers and a higher maximum number of iterations. However, when maximum iterations were set to smaller values, the small-layer QAOA structure performed better. (3) By analyzing the proportions of experimental parameters where $z_{*}$ was an optimal IDS or an IDS (not necessarily optimal), we found that the QAOA showed no significant preference for the number of layers in these indicators. Additionally, by comparing these results with the results from (2), we highlighted the different scenarios in which these two types of indicators are applicable. In the same section, we also performed a complexity analysis. We confirmed that the QAOA-based algorithm offers advantages in terms of complexity compared to other algorithms for solving the IDP. Based on the results from fundamental testing, robustness testing, and complexity analysis, we conclude that using the QAOA to solve the IDP is effective and offers advantages in complexity. However, the QAOA-based algorithm cannot guarantee correct or optimal solutions for the problem under arbitrary parameter combinations, which remains a limitation of the algorithm.

As a supplementary note to this work, the experiments presented in this paper used 10 qubits. As the number of vertices in the graph increases, the required number of qubits may also increase. Although we did not test graphs requiring 20, 30, or more qubits due to experimental constraints, we can offer some insights into situations involving larger numbers of qubits. First, considering the analysis in Figure 9 and Figure 10 and acknowledging that the theoretical performance of the QAOA is layer-dependent [17], we believe that for large-scale qubit systems, increasing the number of layers would theoretically enable achieving the probability levels demonstrated in this paper for both optimal and correct probabilities. However, in light of the complexity analysis and the parameter tendency analysis from the experimental section, it is likely that large-scale qubit systems with a large-layer QAOA would require more iterations. Additionally, a larger number of qubits would impose significant memory demands on local quantum simulations. More qubits also present a broader challenge for quantum computing in general. We note that Zhou et al. [30] addressed the Max-Cut problem by partitioning the graph and applying the QAOA to solve the problem on each subgraph. We believe that this approach could inspire solutions for solving the IDP with large-scale qubits. The study of graph partitioning, graph symmetry, and their intersection with the IDP will be highly beneficial for the further development of IDP solving on quantum computing platforms.

The limitations of this paper include the following: (1) We did not conduct tests of the QAOA for solving the IDP on a quantum computer. (2) We did not optimize the quantum circuits used in this study; instead, we utilized circuits generated by IBM’s Qiskit package.

In the future, based on the work presented in this paper, our research can be extended in the following directions: (1) testing the performance of the QAOA for solving the IDP on real quantum computers; (2) exploring QAOA parameter ranges that are more suitable for the IDP; (3) exploring the relationship between graph partitioning and the IDP; (4) applying the QAOA to solve other variants of the DP, such as the total DP, the perfect DP, and so forth.

## Figures and Tables

**Table 1:** Explanation of components of Equation (16).

Component	Description
$x_{0}+x_{1}+x_{2}+x_{3}+x_{4}+x_{5}$	Size of IDS.
$P\cdot \left(1-x_{0}-x_{2}+x_{0}x_{2}\right)$	Penalty for Constraint (2) of node 0 and N[0]={0,2}.
$P\cdot \left(1-x_{1}-x_{3}+x_{1}x_{3}\right)$	Penalty for Constraint (2) of node 1 and N[1]={1,3}.
$P\cdot {\left(x_{2}+x_{0}+x_{4}+x_{3}-\left(x_{6}+2x_{7}\right)-1\right)}^{2}$	Penalty for Constraint (2) of node 2 and N[2]={0,2,3,4}.
$P\cdot {\left(x_{3}+x_{1}+x_{2}+x_{5}-\left(x_{8}+2x_{9}\right)-1\right)}^{2}$	Penalty for Constraint (2) of node 3 and N[3]={1,2,3,5}.
$P\cdot \left(1-x_{4}-x_{2}+x_{4}x_{2}\right)$	Penalty for Constraint (2) of node 4 and N[4]={2,4}.
$P\cdot \left(1-x_{5}-x_{3}+x_{5}x_{3}\right)$	Penalty for Constraint (2) of node 5 and N[5]={3,5}.
$P\cdot x_{0}x_{2}+P\cdot x_{1}x_{3}+P\cdot x_{2}x_{4}+P\cdot x_{2}x_{3}+P\cdot x_{3}x_{5}$	Penalty for Constraint (4).

**Table 2:** The values of correct and optimal probabilities when q=15, alpha=0.3, P=4.5, and maximum iterations = 10,000.

Correct	Optimal
0.197	0.159

**Table 3:** The comparison of correct and optimal probabilities of different maximum iterations when q=15, alpha=0.3, and P=4.5.

	Probability	Correct	Optimal
Maximum Iterations			
100		0.100	0.057
500		0.144	0.088
1000		0.149	0.094
10,000		0.197	0.159

**Table 4:** The comparison of correct and optimal probabilities of different punishment coefficients when q=15, alpha=0.3, and maximum iterations = 10,000.

	Probability	Correct	Optimal
Punishment Coefficient			
P=3		0.098	0.067
P=4.5		0.197	0.159
P=6		0.196	0.135
P=9		0.176	0.102

**Table 5:** The comparison of the time complexity of algorithms for the IDP.

Algorithm	Time Complexity	Support Quantum Computer?
QAOA	O[poly(q)+poly(m)]	Yes
Brandstädt and Kratsch [5]	$O[|V{|}^{2}]$	No
Breu and Kirkpatrick [6]	$O[|V{|}^{2.376}]$	No
Liu et al. [7] (for (k,l)-graphs)	$O[3.3028^{k}+|V|]$	No
Johnson et al. [8]	$O[2^{0.529|V|}]$	No
Gaspers and Liedloff [9]	$O[2^{0.441|V|}]$	No
Bourgeois et al. [10]	$O[2^{0.424|V|}]$	No

## Data Availability

The data used to support the findings of this study are included within the article.

## Abbreviations

The following abbreviations are used in this manuscript:

DP	Domination Problem
DS	Dominating set
IDP	Independent Domination Problem
IDS	Independent dominating set
QUBO	Quadratic Unconstrained Binary Optimization
QAOA	Quantum Approximate Optimization Algorithm
CVaR	Conditional Value-at-Risk
